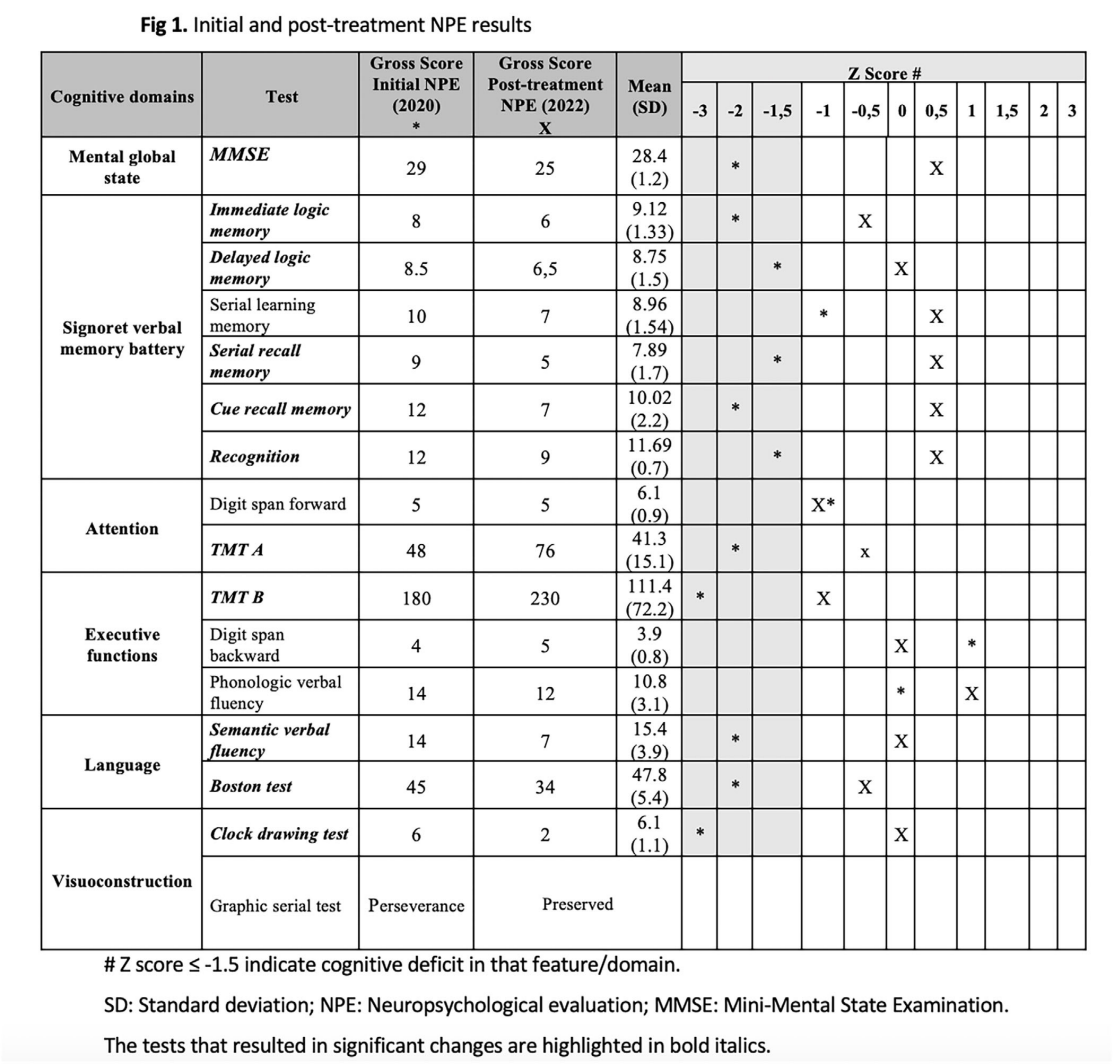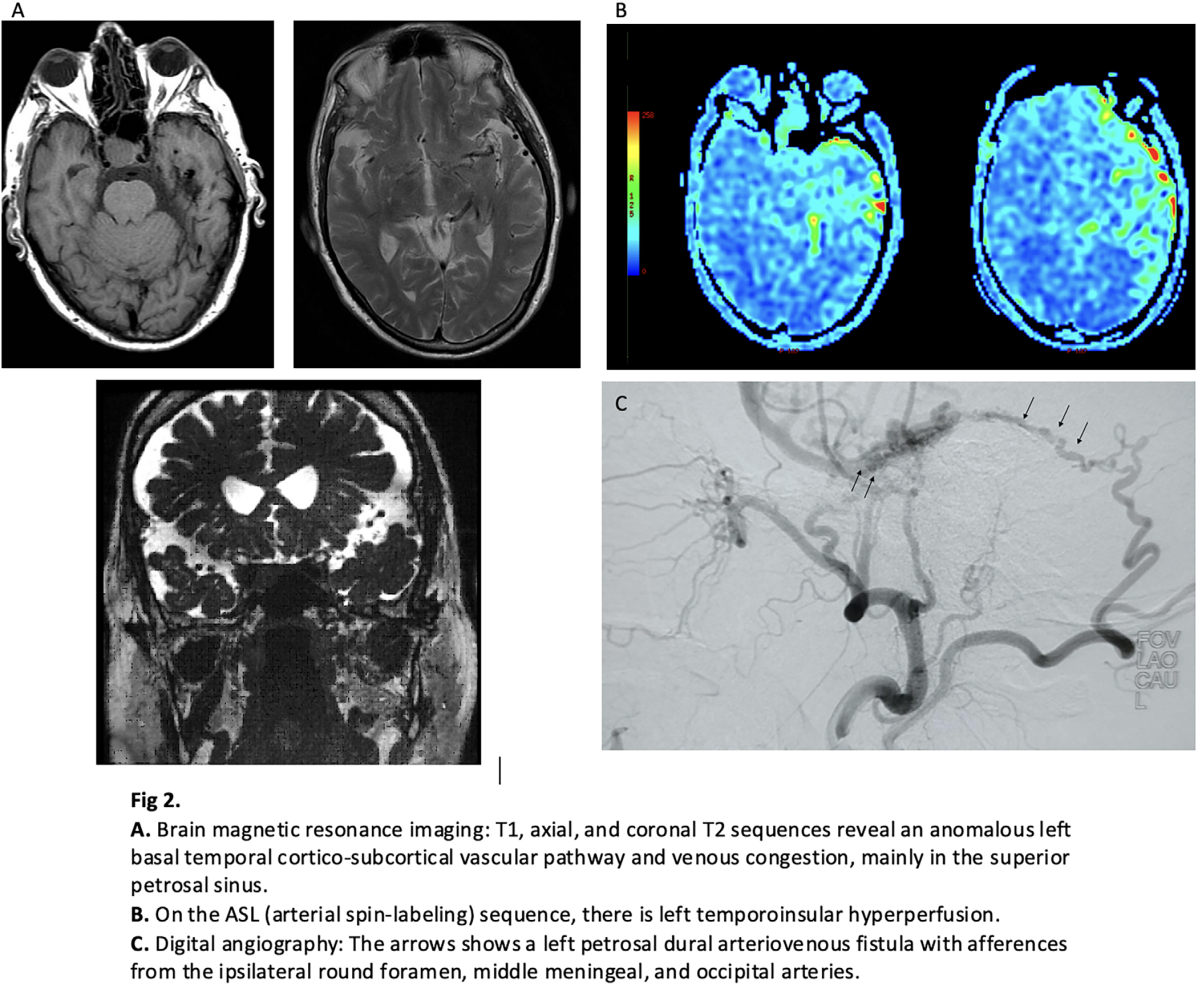# Dural arteriovenous fistula as a cause of reversible cognitive impairment. Case report and a literature review

**DOI:** 10.1002/alz.085985

**Published:** 2025-01-03

**Authors:** Julian Fernandez Boccazzi, Xavier Merchan del Hierro, Barbara Eizaguirre, Galeno Rojas, Adriana Leis, Ramiro Sayavedra, Gabriel Gustavo Persi, Jonathan Cubas Guillen, Victoria Aldinio, Emilia Gatto

**Affiliations:** ^1^ Institute of Neuroscience Favaloro Foundation, Buenos Aires Argentina; ^2^ Sanatorio de la Trinidad Mitre, Buenos Aires Argentina

## Abstract

**Background:**

Dural arteriovenous fistulas (DAVFs) are abnormal communications between dural arteries and cortical, meningeal, or dural sinus veins. They represent 10‐15% of intracranial arteriovenous malformations. In rare cases, they have been associated with potentially reversible cognitive impairment and dementia.

**Method:**

This report presents a clinical case of reversible cognitive impairment associated with DAVF and a review of the literature. Literature research was conducted using PubMed with the following keywords: “Dementia AND Dural arteriovenous fistula”; “Reversible dementia AND dural arteriovenous fistula” and “Dural arteriovenous fistula AND cognitive impairment”.

**Result:**

A case report was presented on a 72‐year‐old male patient who was hypertensive, a smoker, and had a history of headaches. The patient consulted for memory loss that had been ongoing for 3 years and had significantly progressed in the last year. The memory loss was associated with mild anomic aphasia and postural instability. During the examination, the patient exhibited impaired delayed recall and attention. The total score on the Mini‐Mental State Exam (MMSE) was 25 out of 30, and the clock drawing test was failed. The neuropsychological evaluation (NPE) revealed deficits in language, verbal memory, attention, executive functioning, and visuoconstruction. The magnetic resonance imaging (MRI) showed an unusual vascular pathway in the left basal temporal cortico‐subcortical region, with venous congestion mainly in the superior petrosal sinus and hyperperfusion in the left temporal‐insular area in the arterial spin‐labeling (ASL) sequence. Digital angiography confirmed the diagnosis of left petrosal DAVF with afferents from the ipsilateral round foramen, middle meningeal, and occipital arteries. The fistula was completely embolized, and the patient had a favorable outcome. The post‐treatment NEP demonstrated significant improvement, resulting in the normalization of all previously deficient cognitive domains.

**Conclusion:**

While cognitive manifestations secondary to DAVFs are rare, they should be considered as a potentially treatable cause of cognitive impairment. Early identification and adequate treatment have a favorable prognosis and usually lead to a reversal of symptoms, improving the patient’s quality of life.